# Activated Erk Is an Early Retrograde Signal After Spinal Cord Injury in the Lamprey

**DOI:** 10.3389/fnins.2020.580692

**Published:** 2020-11-05

**Authors:** Li-Qing Jin, Brittany H. John, Jianli Hu, Michael E. Selzer

**Affiliations:** ^1^Shriners Hospitals Pediatric Research Center, Lewis Katz School of Medicine, Temple University, Philadelphia, PA, United States; ^2^Department of Neurology, Lewis Katz School of Medicine, Temple University, Philadelphia, PA, United States

**Keywords:** spinal cord injury, axon, retrograde signal, Erk, c-Jun, dynein, vimentin

## Abstract

We previously reported that spinal cord transection (TX) in the lamprey causes mRNA to accumulate in the injured tips of large reticulospinal (RS) axons. We sought to determine whether this mRNA accumulation results from phosphorylation and transport of retrograde signals, similar to what has been reported in mammalian peripheral nerve. Extracellular signal-regulated protein kinase (Erk), mediates the neurite outgrowth-promoting effects of many neurotrophic factors. To assess the role of Erk in retrograde signaling of RS axon injury, we used immunoblot and immunohistochemistry to determine the changes in phosphorylated Erk (p-Erk) in the spinal cord after spinal cord TX. Immunostaining for p-Erk increased within axons and local cell bodies, most heavily within the 1-2 mm closest to the TX site, at between 3 and 6 h post-TX. In axons, p-Erk was concentrated in 3-5 μm granules that became less numerous with distance from the TX. The retrograde molecular motor dynein colocalized with p-Erk, but vimentin, which in peripheral nerve was reported to participate with p-Erk as part of a retrograde signal complex, did not colocalize with p-Erk, even though vimentin levels were elevated post-TX. The results suggest that p-Erk, but not vimentin, may function as a retrograde axotomy signal in lamprey central nervous system neurons, and that this signal may induce transcription of mRNA, which is then transported down the axon to its injured tip.

## Introduction

Cells have evolved signaling systems that identify environmental challenges, and relay the information to the nucleus, where protective and regenerative genetic responses are initiated. Transcription factors typically are located close to the nucleus, but injury to an axon might require signals to be transported retrogradely for long distances to the nucleus, in order to elicit transcriptional programs (Ambron and Walters, 1996). The mitogen-activated protein kinase (MAPK) pathways have been implicated in neuronal responses to many extracellular stimuli, including peptide growth factors, cytokines, and axotomy. These signaling pathways regulate cellular responses, including proliferation, differentiation, survival, and death (Kim and Choi, 2010), through activation of transcription factors. The basic assembly of MAPK pathways is a three-component module composed of MAPK kinase (MKKK), MAPK kinase (MKK), and MAPK.

Extracellular signal-regulated kinase (Erk), one of four conventional MAPKs (Cargnello and Roux, 2011), has been implicated as an initial signal of injury in neuronal apoptosis (Cheung and Slack, 2004), neurite outgrowth (Huang et al., 2017), and cell proliferation (Zhang and Liu, 2002). Upon injury in mammalian peripheral nerve, Erk is phosphorylated (p-Erk) in the axoplasm, and in some axons, has been reported to form a retrograde signal complex with a vimentin fragment and importin-β, and then trafficked retrogradely on dynein motors (Perlson et al., 2005). In some neurons, injury is signaled by other pathways (Rishal and Fainzilber, 2014). Unfortunately, the complexity of the central nervous system (CNS) in most vertebrates imposes significant constraints on extending knowledge about the responses of neurons to axon injury from peripheral nerve to the CNS *in vivo*. For example, mammalian models of spinal cord injury (SCI) rely largely on incomplete lesions, making it difficult to distinguish true axon regeneration from collateral sprouting by spared axons. The mechanisms underlying these two processes appear to be different, and molecular manipulations that increase sprouting may not affect regeneration (Lee et al., 2009). The responses of neurons to axotomy, *e.g.*, retrograde neuronal death and axon regeneration, are typically studied in mice because of their advantages for genetic manipulation. But mice are so small that the distances of axon growth required to form synaptic relays that can mediate functional recovery might be within the sprouting range. It is likely that most of the molecular manipulations that have been reported to promote functional recovery in rodents actually enhanced sprouting, but may not have enhanced regeneration of injured axons (Lee et al., 2009). In order to get around these limitations, some laboratories have used non-mammalian species, such as the lamprey, a primitive vertebrate that shared a common ancestor with humans about 550 million years ago (Smith et al., 2013). Axons in the lamprey CNS can regenerate, but this is incomplete, both with regard to distance and to the probability that an individual neuron will survive axotomy and regenerate its axon; some neurons are very good regenerators and survivors, and other neurons not. Thus there is room for experimental manipulations to make regeneration and neuronal survival better or worse. Because of several other advantages, such as translucency of the CNS, the presence of large identified reticulospinal (RS) neurons, and the ability to study the CNS in isolated preparations, the lamprey has served as an important model to study spinal cord injury and axon regeneration (Rovainen, 1976; Selzer, 1978; McClellan, 1988; Jin et al., 2009, 2016; Lau et al., 2013; Hanslik et al., 2019). The giant Müller and Mauthner cells of the brainstem give rise to giant axons, ranging from 20 to 40 μm in diameter. These course in stereotypic paths the length of the spinal cord (Rovainen, 1967), and can be readily impaled with sharp electrodes for physiological studies (Selzer, 1979; Cochilla and Alford, 1997; Photowala et al., 2005). The genome of the sea lamprey has been sequenced (Smith et al., 2013, 2018), although incompletely annotated, and contains homologs of almost all of the mammalian gene families, including many suspected of influencing axon regeneration (Shifman and Selzer, 2000a, b, 2006; Shifman et al., 2009; Jin et al., 2011; Lau et al., 2011; Zhang et al., 2014; Chen et al., 2015; Herman et al., 2018). RNAseq of CNS after spinal cord TX in lampreys has suggested that many pro-regeneration mechanisms are evolutionarily conserved, *e.g.*, the Wnt signaling pathway (Herman et al., 2018).

In the current study, we show that Erk is activated early at the injury site, and that p-Erk is translocated retrogradely in granules, colocalized with dynein but not vimentin. In mammals and most other tetrapods, there are two very similar Erk proteins, Erk1 and Erk2 (Boulton et al., 1991), which are products of two closely related genes. Because they have greatly overlapping functions and are recognized by most available anti-Erk antibodies, they are commonly referred to as Erk1/2. Thus far, only one Erk gene has been recognized in the (incompletely annotated) lamprey genomic library (Smith et al., 2013, 2018), and only one Erk protein has been detected (weakly) on Western blots of lamprey CNS (Buscà et al., 2015). We now verify the existence of only one lamprey Erk by browsing the gene in a recently assembled lamprey germline cell genome (Pmar_germline 1.0/petMar3) and probing lamprey CNS homogenates by immunoblot.

## Materials and Methods

### Animal and Spinal Cord Transection

A total of 222 larval lampreys (*Petromyzon marinus*) 10–13 cm in length (4-5 years old and in a stable phase of neurological development) were purchased from Lamprey Services in Michigan, or gifted (100) by Dr. Nicholas Johnson of the USGS Great Lakes Science Center, and maintained with light cycle in fresh water tanks at 15°C until use. All animal procedures were performed with a protocol approved by the Temple University Institutional Animal Care and Use Committee (ACUP#: 4609). Lampreys were anesthetized by immersion in saturated aqueous benzocaine for 5 min and pinned to a Sylgard (184 silcone elastomer, Dow Corning) plate filled with ice-cold lamprey Ringer (110 mM NaCl, 2.1 mM KCl, 2.6 mM CaCl_2_, 1.8 mM MgCl_2_, and 10 mM Tris buffer; pH 7.4). The spinal cord was exposed *via* a dorsal incision and transected under direct microscopic vision with iridectomy scissors at the level of the 7th gill unless otherwise specified. For most acute tests, animals were either untransected (controls) or kept on ice for 2 h after TX, exposed to air to facilitate clot formation. Thereafter, animals were returned to fresh water tanks at 4°C and sacrificed at 3 or 6 h post-TX, or allowed to recover at 4°C overnight.

### Cryostat Sectioning and Immunohistochemistry (IHC)

To avoid injury signals generated during the isolation of tissues, all lampreys in control groups were killed instantly by freezing on a metal plate that was pre-cooled on dry ice. A length of body containing the brain and spinal cord rostral to the TX site was obtained by cutting the frozen tissue at the olfactory sac in the head and 1 mm below 7th gill in the spinal cord. The head was separated from the spinal cord by a third cut at locations specified in section “Results.” The frozen stumps were placed in stainless steel molds (10 × 10 × 10 mm, Simport Scientific) filled with Optimal Cutting Temperature (OCT) medium. The orientation of the frozen tissue was adjusted to obtain transverse or coronal (horizontal) sections. The molds were placed directly on top of liquid nitrogen in a Dewar flask until the OCT became completely solid. Blocks were stored at −80°C until sectioning. Cryosection was performed on a Leica CM1950 Cryostat with chamber temperature −17°C. The frozen blocks were placed in a cryochamber for 30 min to achieve uniform temperature before sectioning. The OCT-embedded specimens were cut serially at 15 μm and mounted on glass slides. Slides were kept temporarily in a Styrofoam box filled with dry ice during sectioning, and then stored at −80°C until use.

For IHC, slides were thawed and dried in a plastic box filled with Drierite desiccant (Drierite Co.) at 4°C for 15 min, and then at room temperature for 30 min. The tissue was fixed with ice-cold acetone for 2 min and washed with phosphate-buffered saline (PBS) twice for 30 s. Non-specific binding was blocked with 4% fetal bovine serum (FBS, in PBS, pH 7.5) for 5 min. Slides were incubated with various antibodies (Table 1) overnight at 4°C. The next day, samples were washed in Tris-buffered saline solution (TBS, 0.1% Tween 20 in PBS, pH 7.5) and blocked by normal animal serums depending on the origin of the secondary antibodies. For immunofluorescence, slides were incubated with either Alexa Fluor 488 donkey anti-mouse IgG polyclonal antibody or Alexa Fluor 594 donkey anti-rabbit IgG polyclonal antibody (Table 1) at 1:200 in blocking solution for 1 hour at room temperature. Finally, the specimens were washed twice with TBS and mounted with DAPI Fluoromount-G (Southern Biotech, 0100-20). Images were captured using a Nikon Eclipse 80i epifluorescence microscope with a Roper Scientific CoolSNAP digital camera, under the control of NIS Elements software. Negative controls employed the same protocol, omitting the primary antibody. For chromogenic IHC, protocols recommended by Cell Signaling Technology were followed. It generates brown to gray-black colors.

**TABLE 1:** Primary and secondary antibodies.

Name	Host species and antigen	Manufacturer	Dilution	RRID
Anti-vimentin (clone V9)	Mouse mAb	Sigma-Aldrich	1:1,000 (WB) 1:2,00 (IHC)	AB_477627
LCM16 (anti-NF180)	Mouse, lamprey CNS cytoskeletal protein	Author’s lab	1:100 (IHC)	AB_2861400
IRDye@800CW	Goat anti-mouse IgG	LI-COR	1:7,500 (WB)	AB_621842
IRDye@680RD	Goat anti-rabbit IgG	LI-COR	1:7,500 (WB)	AB_621841
Phospho-p44/42 MAPK (Erk1/2) (Thr202/Tyr204)	Rabbit mAb	Cell Signaling Technology	1:1,000 (WB) 1:1,00 (IHC)	RRID: AB_2315112
p44/42 MAP Kinase (L34F12)	Mouse mAb	Cell Signaling Technology	1:1,000 (WB) 1:1,00 (IHC)	AB_390780
Phospho-c-Jun (Ser63) II	Rabbit pAb	Cell Signaling Technology	1:1,000 (WB)	AB_2130162
Anti-Dynein (clone 74.1)	Mouse mAb, 74 kDa Intermediate chains	Sigma Aldrich Incorporated	1:1,000 (WB) 1:1,00 (IHC)	AB_2246059
Alexa Fluor 488	Donkey anti-mouse IgG H&L	Abcam Ltd.	1:2,00 (IHC)	AB_2732856
Alexa Fluor 594	Donkey anti-rabbit IgG	BioLegend	1:2,00 (IHC)	AB_2563306

### Molecular Cloning of the Lamprey Vimentin Full-Length Gene

A cDNA fragment (717 bp) by-product of PCR that was performed with the goal of cloning new NF proteins (Jin et al., 2011) was highly homologous to the vimentin genes of other species. Six primers were designed to clone the full length of this intermediate filament by PCR, using a lamprey Uni-ZAP XR cDNA library as a template. This vimentin gene (L-vimentin) consists of 3380 nucleotide bp and has been deposited in GenBank (HQ171987).

### *In situ* Hybridization With Biotin-Labeled Riboprobes

Hybridization of biotin-labeled riboprobes to paraffin sections of lamprey spinal cord was performed as reported previously (Swain et al., 1994). Briefly, the anesthetized lamprey was killed instantly on dry ice and fixed in 4% paraformaldehyde at room temperature overnight. The sample was washed in PBS, and a 7mm length of lamprey body including the 7th gill was isolated. This piece was dehydrated in serial ethanols, cleared in toluene, infiltrated with Paraplast, and embedded in paraffin. Transverse 10 μm paraffin sections were collected, deparaffinized, and rehydrated in xylene and serial ethanols.

#### Vimentin Riboprobes

A T7-tagged cDNA fragment was obtained by PCR, using a pair of primers (primer A, 5′-TAAGCTTTAATACGACTCACTATAGGGAGAGGATTTTCT TGATTATGTTGG-3; primer B, 5′-CGTGTACAGCAAGAA GACTGTGCTCATCAAGACGATTGAGACG-3′), and a cloned lamprey vimentin cDNA as template. The primers cover nucleotides 1276-1696 of the gene (421 bp), a region just after the stop codon. Another pair of primers (C, 5′-TAAGCTTTAATACGACTCACTATAGGGAGACGTGTACAG CAAGAAGACTGTGC-3′; D, 5′-GGATTTTCTTGATTAT GTTGGTTGTGAGAATTCTTAAACTCACC-3′) were used for PCR amplification of the same regions with a T7 tag on the other strand of gene. T7 RNA polymerase was used for sense or antisense probe synthesis. Digoxigenin-labeled riboprobes were synthesized *in vitro*, using an RNA transcription kit (Roche, Nutley, NJ, United States) as per the manufacturer’s instructions.

Sectioned tissues were prehybridized at 55°C in hybridization solution (50% deionized formamide, 5 × SSC, 100 pg/ml *Torula* yeast RNA, 100 pg/ml wheat germ tRNA, 50 pg/ml heparin, 0.1% Tween-20), followed by hybridization overnight at 55°C in the same solution plus 2 μg/ml digoxigenin-labeled cRNA. Specimens were washed in hybridization solution, PBS-Tween solution (PTw, 0.1% Tween-20 in PBS), and PBS-FBS-Triton solution (PBT, 0.1% FBS, 0.2% Triton X-100 in PBS). An alkaline phosphatase-conjugated sheep anti-digoxygenin Fab fragment (1:1000) was applied to the slides for 3 h at room temperature. Specimens were washed sequentially in PBT and Sodium-Magnesium-Tween solution (SMT, 100 mM NaCl, 50 mM MgCl_2_, 100 mM Tris, pH 9.5, 0.1% Tween-20). The chromogenic reaction was carried out in ice-cold SMT containing 175 pg/ml 5-bromo-4-chloro-3-indolyl-phosphate and 350 pg/ml 4-nitro blue tetrazolium chloride for 1-2 h in the dark at room temperature. Finally, slides were washed in PBS, dehydrated in serial ethanols, cleared with xylene, and mounted under Permount (Thermo Fisher scientific). Stained samples were visualized on a Zeiss Axiovert 135 microscope using 10 or 20× dry objectives. Images were acquired using a Zeiss Hi Resolution color camera and software (Zeiss Axioskop).

### Western Blotting

**A, Vimentin.** A total of 48 *Petromyzon marinus* specimens were anesthetized in saturated aqueous benzocaine, and their brains and spinal cords were dissected. Tissues were homogenized by Ultrasonic Processor (FB120, Thermo Fisher Scientific) in homogenization buffer consisting of 25 mM HEPES (pH 7.4), 100 mM sucrose, 1 mM EGTA, 0.2% β-mercaptoethanol, 0.04 mM PMSF, 50 μg/ml leupeptin, 25 μg/ml pepstatin A, and 0.01 U/ml soybean trypsin inhibitor. The homogenate was centrifuged at 800 × *g* for 10 min at 4°C. A first supernatant was collected and recentrifuged at 10,000 × *g* for 15 min. The second pellet was enriched in cytoskeleton, and used for vimentin assay. **B, Erk** and **p-c-Jun**, forty-eight brains and spinal cords were dissected and homogenized in RIPA buffer (Pierce) containing 20 mM Tris-HCl (pH 7.5), 150 mM NaCl, 1 mM Na_2_EDTA, 1 mM EGTA, 1% NP-40, 1% sodium deoxycholate, 2.5 mM sodium pyrophosphate, 1 mM β-glycerophosphate, 1 mM Na_3_VO_4_, and 1 μg/ml leupeptin, by sonication. Whole homogenate was collected and used for Western blotting. Protein concentrations were quantified by the Lowry method. Proteins (35 μg) were separated in a 10% SDS mini-gel, and were electrophoretically transferred onto nitrocellulose membranes (0.45 μm, Bio-Rad) using a Bio-Rad transblot apparatus. The membranes were blocked in Odyssey Blocking Buffer (LI-COR, 927-40000) for 2 h and incubated with antibodies (Table 1) overnight at 4°C, followed by washing with PBS containing 0.2% Tween 20 (PBST), and incubation with the fluorescence labeled secondary antibody (Table 1) for 1 h at room temperature. The membranes were then rinsed in PBST and scanned by the Odyssey infrared imaging system (LI-COR Biosciences, Lincoln, NE, United States).

### Quantification and Statistical Analysis

#### IHC

Surrounding background and meningeal staining in spinal cord sections were removed by Adobe Photoshop (CS4). Quantification was performed using image analysis software ImageJ (1.52a). Image was converted to 8 bit format. Total area (**TA**, in pixels) was assessed after adjustment of low and high threshold so that whole spinal cord become completely dark. Stained area (**SA**) was quantitated after adjustment of high threshold to the point that the image is identical to the pattern of real staining. The high threshold value for control group was used for other groups. The ratio between SA and TA is defined as “percent intensity” for each image. This process was repeated twice for each section to ensure that the measurement was consistent.

#### Western Blotting

All quantifications were processed in ImageJ software as described (Gallo-Oller et al., 2018). Briefly, images were converted to 8-bit format in order to perform uncalibrated optical density (OD). After conversion, the background was subtracted through bright/contrast adjustment. Each band was individually selected and circumscribed with the rectangular ROI selection and “Gels” function, followed by quantification of peak area of obtained histograms. Data were acquired as arbitrary area values.

#### Statistical Analysis

All data are represented as the mean ± standard deviation for all performed repetitions. Once normality and homogeneity of variance analysis were assessed, a parametric or non-parametric analysis was selected. Statistically significant differences among three or more groups were analyzed by one-way analysis of variance (ANOVA), followed by *post hoc* analysis of Tukey or Dunnett, respectively. Student’s *t*-test was used for significant difference between 2 groups. Correlation analysis was performed using linear regression to get Pearson’s r coefficient. The significance level was set to *p* < 0.05 and classified by asterisks as follows: *p* < 0.05 (^∗^); *p* < 0.01 (^∗∗^); *p* < 0.001 (^∗∗∗^). The statistical analysis was calculated through SigmaStat software for Windows (version 2.0). Sample numbers were indicated in individual legend.

## Results

### The Lamprey Lacks *erk1* Genes

**FIGURE 1:**
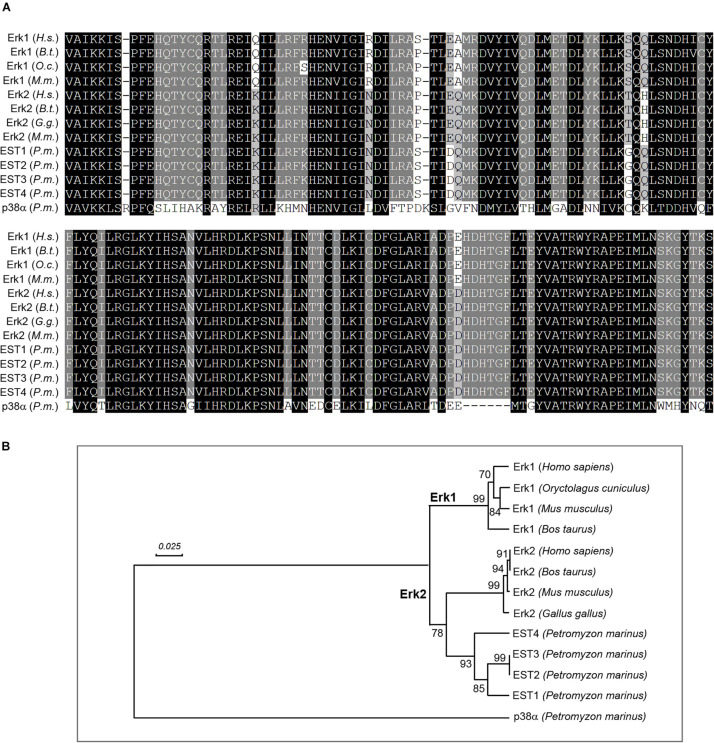
Lamprey lacks Erk1 isoforms. **(A)** alignment of lamprey and mammalian Erk protein sequences. Thirteen DNA sequences (1 lamprey cDNA, 4 lamprey ESTs, and 8 mammalian cDNAs) were converted to protein sequences and aligned with ClustalX software. The generated MSF file was edited with GeneDoc. Conserved amino acid residues are shaded black. Conservative substitutions are in gray. The two most-conserved blocks (158 amino acids) are presented. **(B)** positions of lamprey **Erks** in a phylogenetic tree constructed with human, cattle, rabbit, chicken, and mouse Erks using the “UPGMA” algorithm. Four lamprey protein sequences (EST1-EST4), converted from corresponding *erk* EST sequences (cDNA), were aligned with 8 mammalian Erks using MEGA-X software (MUSCLE). Four lamprey Erks corresponding to EST sequences CO544767, CO548040, CO545630 and CO547047 were within the **Erk2** clade. No lamprey genes were grouped under the **Erk1** subtree. Numbers to the left of the branch points indicate the percent of 10,000 bootstrap replicates that support that branch. Lengths in the tree reflect distances between taxa (mean substitutions per residue). Lamprey p38α was chosen to provide an outgroup for rooting the Erk tree. Scale: 0.025 amino acid substitutions per site.

In studying the involvement of Erk1/2 in retrograde signaling, a related question is whether there is only one Erk isoform in lamprey, as described previously (Buscà et al., 2015). A recent assembly of the lamprey genome has been reported (Smith et al., 2018). As compared with its earlier version (Smith et al., 2013), which was constructed from somatic tissues, the newly assembled lamprey genome is more complete because it was developed from germline cells, and will not undergo rearrangements during early embryogenesis, in which approximately 20% of the germline DNA is shed (Smith et al., 2009). Searching the lamprey genomic database (Pmar_germline1.0/petMar3) with “Erk” generated 8 lamprey EST sequences (ESTs) including CO544767, CO545630, CO547047, CO548040, CO548101, DW585863, EG025241, and FD719223. Three of them (CO548101, DW585863, and EG025241) have been removed in construction of a phylogenetic tree because of their short nucleotide and amino acid lengths (Table 2). Another lamprey EST, FD719223 was outside of the MAPK group, and turned out to be a MAPKK protein by BLAST searching of the NCBI database. Eight mammalian genes coding for ERK1 (NM_002746, NM_001110018, XM_008257945, and NM_011952) and ERK2 (NM_002745, NM_175793, XM_015275131, and NM_001357115) were obtained by searching the NCBI database^1^ with “Erk1,” and “Erk2” (Table 2). Another lamprey gene coding for p38α protein (XM_032969733) was used as an outgroup node for rooting the Erk tree (Cargnello and Roux, 2011). The most homogeneous region (158 aa) in 13 sequences is presented in Figure 1A, which covers Exon-2 ∼ Exon-5 regions. A phylogenetic tree was constructed from 11 full length and 2 partial length protein sequences (Table 2) by MEGA-X software (Kumar et al., 2018; Figure 1B). Four lamprey ESTs belong to the Erk2 clade. No lamprey sequence fell into the Erk1 category. Identity within mammalian Erk1 is 89.5-96.5%, whereas within mammalian Erk2 it is 95.9-99.7%. Identity between mammalian Erk1 and Erk2 is 78.6-85.4%. Lamprey Erk2 and mammalian Erk2 were 54.6-68.8% identical. These results indicate that the lamprey lacks Erk1 isoforms. This is consistent with the single band seen in Western blotting with antibodies that in mammals, recognize both Erk1 and Erk2. Therefore, in referring to the lamprey version of this protein, we use only the term “Erk.”

**TABLE 2:** DNA sequences selected in constructing an *erk* phylogenic tree.

Name	Access No.	n.t.	AA length (P/F)*
Erk1 (*Homo sapiens*)	NM_002746.3	1787	379 (F)
Erk1 (*Bos taurus*)	NM_001110018.1	2135	362 (F)
Erk1 (*Oryctolagus cuniculus*)	XM_008257945	1758	393 (F)
Erk1 (*Mus musculus*)	NM_011952.2	1772	380 (F)
Erk2 (*Homo sapiens*)	NM_002745.5	5881	360 (F)
Erk2 (*Bos taurus*)	NM_175793.2	1272	360 (F)
Erk2 (*Gallus gallus*)	XM_015275131.2	10028	368 (F)
Erk2 (*Mus musculus*)	NM_001357115.1	5094	358 (F)
p38α (*Petromyzon marinus*)	XM_032969733.1	4017	359 (F)
EST1 (*Petromyzon marinus*)	CO544767	934	> 310 (P)
EST2 (*Petromyzon marinus*)	CO545630	795	> 262 (P)
EST3 (*Petromyzon marinus*)	CO547047	928	262 (F)
EST4 (*Petromyzon marinus*)	CO548040	823	242 (F)
EST5 (*Petromyzon marinus*)**^×^**	CO548101	552	> 58 (P)
EST6 (*Petromyzon marinus*)**^×^**	DW585863	211	> 69 (P)
EST7 (*Petromyzon marinus*)**^×^**	EG025241	478	69 (F)
EST8 (*Petromyzon marinus*)**^×^**	FD719223	736	> 182 (P)

^×^Sequence not used in the phylogenic tree (see Figure 1B). Only 13 of the sequences were used (See text for explanation). *F, full length (start codon and stop codon are included); P, partial length (either the start codon or the stop codon is not included).

### IHC in Transverse Sections of Transected Lamprey Spinal Cord

Although the lamprey spinal cord is relatively simple, and translucent under a dissecting microscope, it is still difficult to monitor the transport of a labeled protein along spinal cord axons in the living animal. Thus, we resorted to immunofluorescence labeling and chromogenic immunohistochemistry at different locations and times, to deduce the movement of immunolabeled p-Erk inside the axon. Animals were killed in less than 1 min by instant freezing on a dry ice-chilled metal plate. This served to minimize any injury signals that might be generated in even control spinal cords within the first seconds to minutes of tissue trimming and fixation (Ambron and Walters, 1996).

### In Untransected Control Animals, p-Erk Content Is Sparse and Restricted to Cell Bodies

**FIGURE 2:**
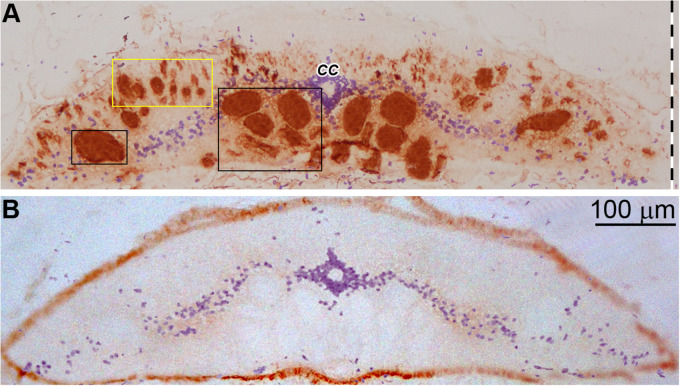
Immunohistochemistry of lamprey spinal cord with LCM16 and p-Erk. **(A)** One spinal cord (body length = 10.5 cm) was sectioned transversely at the 7th gill with a cryostat and stained for neurofilaments with mAb LCM16 (1:100). The slide was stained by SignalStain DAB Chromogen (brown, Cell Signaling Technology) and counter-stained with hematoxylin (blue). Note: LCM16 labels all giant axons and most medium and small caliber axons, but not those in the dorsal columns; hematoxylin labels mostly the nuclei in the gray matter and the ependymal cells surrounding the central canal ***(CC)***. Left-sided giant axons that project to the spinal cord are framed by 2 black rectangles. Small and medium-sized axons are framed by the yellow rectangle. **(B)** Slides from the same animal were probed by anti-p-Erk (1:100, Cell Signaling Technology) and counter-stained with hematoxylin. Note: Little or no p-Erk staining was seen in the giant axons of untransected spinal cord.

A lamprey monoclonal antibody LCM16 was used to determine if an immunolabeled granule is intra-axonal. The antibody is one of 43 LCM series monoclonal antibodies, which were generated previously in this laboratory against cytoskeletal proteins of the lamprey nervous system (Merrick et al., 1995). On Western blots, it is a phosphorylation state-independent antibody against the lamprey neurofilament subunit NF180, and has been used previously in IHC studies (Jacobs et al., 1997; Zhang et al., 2005; Hanslik et al., 2019). Chromogenic IHC with LCM16 is seen in a transverse section of lamprey spinal cord in Figure 2A. The antibody is selective for axons, with very little staining in the neuronal cell bodies of the “gray matter,” whose nuclei are counterstained with hematoxylin. Most of the giant axons that were mapped previously in serial transverse sections (Rovainen, 1982; Buchanan, 2001) can be recognized. Thus, this antibody was used to identify intra-axonal profiles, and distinguish them from other neuronal compartments. Chromogenic IHC labeling with an anti-p-Erk antibody [anti-p44/42 MAPK (Erk1/2), Cell Signaling Technology] in transverse sections of control lamprey spinal cord (Figure 2B) showed no staining in any of the giant axons, but some light staining was seen in the gray matter.

### Spinal Cord TX Increases p-Erk Labeling in Neurons and Glia Near the Injury

**FIGURE 3:**
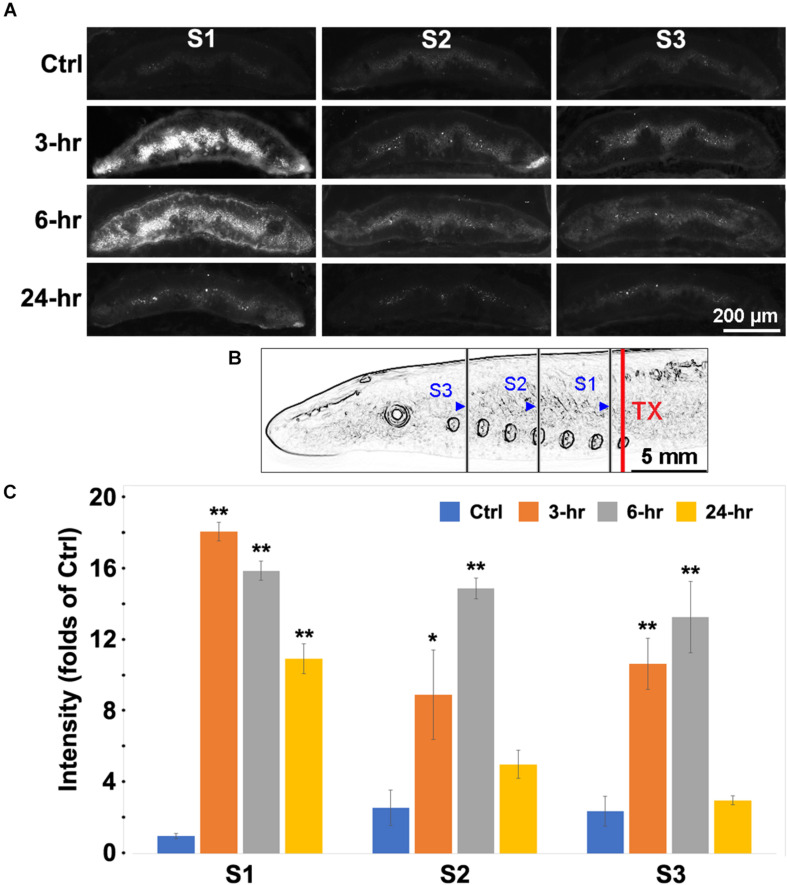
Labeling for p-Erk was increased after spinal cord TX. **(A)** Fluorescent images of transverse sections of spinal cord at locations S1, S2, and S3 were obtained in 4 time groups: No TX (**Ctrl**), **3**, **6**, and **24 h** post-TX. Slides were labeled with a primary antibody against p-Erk, and fluorescently labeled secondary antibody (AF-594). The strongest p-Erk staining was found at 3 and 6 h in gray matter near the TX (C1). **(B)** schematic illustration of the S1, S2, and S3 body locations at which the spinal cord was imaged after TX at the 7th gill. **Blue triangles:** caudal boundaries of the body lengths for transverse sectioning. S1 = the length between the 4th and 6.5th gills; S2 = 1.5th-4th gill; S3 = Head-1.5th gill. **(C)** quantification of p-Erk response to TX. Spinal cord TX significantly increased the p-Erk labeling at 3 and 6 h at all sites. (Mean ± SEM, *n* = 4; *, **p < 0.05/0.01 over Ctrl, respectively, one-way ANOVA followed by Dunnett’s test).

Erk is rapidly phosphorylated in response to multiple stimuli. We measured Erk phosphorylation in cells of the gray matter along the lamprey spinal cord by fluorescence IHC. Animals had spinal cord TX at the level of the 7th gill and survived for 0 (Ctrl), 3, 6, or 24 h. Then their spinal cords were sampled by frozen sections at three locations - between the 6th and 7th gill (6.5th gill), the 4th gill, and between the 1st and 2nd gill (1.5th gill), as illustrated in Figure 3B. The sections were immunolabeled with p-Erk primary and fluorescent secondary antibody. Spinal cord TX greatly increased fluorescence intensity in neurons and glial cells of sections (Figure 3A) at 3 h (17-fold), 6 h (15-fold), and 24 h (10-fold) post-TX near the site of injury (6.5th gill). At locations further from the TX (4th and 1.5th gill), similar responses were observed at 3 h (8-fold and 10-fold) and 6 h (14-fold and 13-fold), respectively. Fluorescence intensity dropped to near control levels by 24 h post-TX at the 4th (4-fold) and 1.5th-gill (2-fold) (Figure 3C). Thus, Erk phosphorylation is prompt and robust in cells close to the TX. We did not attempt to identify the local cells responding to the TX precisely, but by their sizes and locations, it was clear that the cells showing increased p-Erk labeling included both neurons and glia.

### Increased p-Erk Levels Result From the Phosphorylation of Pre-existing Erk

**FIGURE 4:**
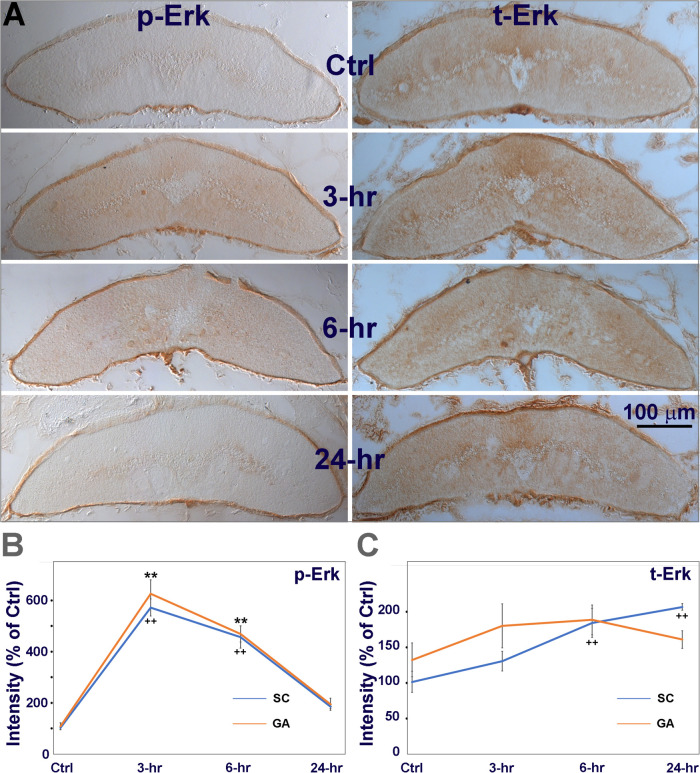
The increase in p-Erk is due mostly to phosphorylation of pre-existing Erk. **(A)** chromogenic IHC for phosphorylated Erk (p-Erk), and total Erk (t-Erk). Animals had a spinal cord TX at the level of the 7th gill. Transverse sections near the TX site (∼6.5th gill) were collected from 4 groups of animals. Animals were sacrificed immediately **(Ctrl)**, or at **3**, **6,** or **24 h post-TX**. **Left**, spinal cord immunolabeled with a p-Erk antibody, **Right**, labeled with a t-Erk antibody. **(B)** Quantification of immunolabeled p-Erk in whole spinal cord (SC) and in giant axons (GA, as defined in Figure 1A). In both SC and GA, Erk was significantly activated (phosphorylated) at 3 and 6 h post-TX (5-6 fold over Ctrl), and returned to low levels by 24 hours. **(C)** total Erk was increased both in SC (1.5∼2 fold) and GA (1.5-fold) (Mean ± SEM, *n* = 6 animals; **, ^++^ *p* < 0.01 over Ctrl in GA/SC, respectively, one-way ANOVA followed by Tukey test). Thus, the increase in t-Erk was too small to account for the large increase in p-Erk.

Next we used chromogenic IHC to determine whether the increase in p-Erk post-TX was due to an increase in Erk expression, or to phosphorylation of pre-existing Erk. Animals were divided into 4 groups as above. The spinal cords were transected at the 7th gill. Transverse sections were made at the 6.5th gill and chromogenic IHC was performed with primary antibodies against p-Erk and total Erk (t-Erk). In control spinal cords, p-Erk levels were very low (Figures 2B, 4A). Spinal cord TX significantly increased p-Erk levels – at 3 h by 5.5-fold; 6 h by 4.4-fold; and 24 h by 1.8-fold (Figure 4B). This also was true when analysis was limited to the ventral columns, where the giant Müller axons course (Figure 4B). IHC for t-Erk on the same sections indicated that increases in t-Erk were much less dramatic, although statistically significant at 6 h (1.78-fold) and 24 h (2-fold). Quantification in the area containing the giant axons showed no significant increases in t-Erk (Figure 4C). This suggests that the increases in p-Erk levels in whole spinal cord or in the giant axons are mostly due to phosphorylation of existing Erk.

### Spinal Cord TX Increases the Number of Intra-axonal p-Erk-Containing Granules

**FIGURE 5:**
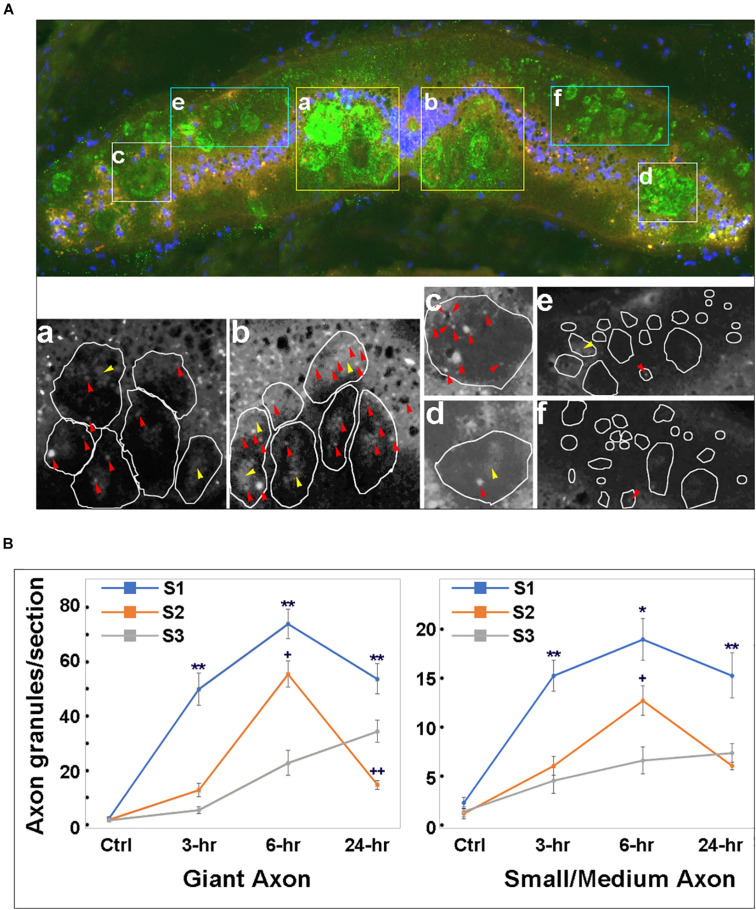
Time course for phospho-Erk distribution in axons of transected spinal cord. **(A) Top panel,** a representative transverse section at S2 level of a spinal cord at 3 h post-TX, stained with a p-Erk pAb (red), LCM16 mAb (green) and DAPI (blue). Axons stained with LCM16 were clustered into 6 regions **(a–f)**. **Bottom panel,** magnified black and white images from the red channel for the 6 regions in the top micrograph, showing p-Erk granules. Irregular circles are axon profiles identified by LCM16 staining. **(a,b)**, granules inside giant axons in the ventral columns. **Red arrowheads** point to isolated granules; **yellow arrowheads** point to diffuse intra-axonal staining. **(c,d)**, granules inside the Mauthner axons in the lateral columns. **(e,f)**, granules inside small-medium axons in dorsolateral columns are less numerous than those in the largest axons. The small axons of the dorsal columns are not sampled because these axons are primarily rostral-projecting and also stain poorly with LCM16, but they too have almost no p-Erk-containing granules. **(B)** at 3, 6, and 24 h post-TX, granule numbers at 3 locations (S1, S2, and S3) are significantly greater than in controls (*p* < 0.01, one-way ANOVA). Granule numbers at S1 of transected spinal cords are significantly greater than those at S2 and S3 (Mean ± SEM, *n* = 10; *, **p < 0.05/0.01 over S2, respectively, ^+,^^++^*p* < 0.05/0.01 over S3, respectively, one-way ANOVA followed by Tukey test).

**FIGURE 6:**
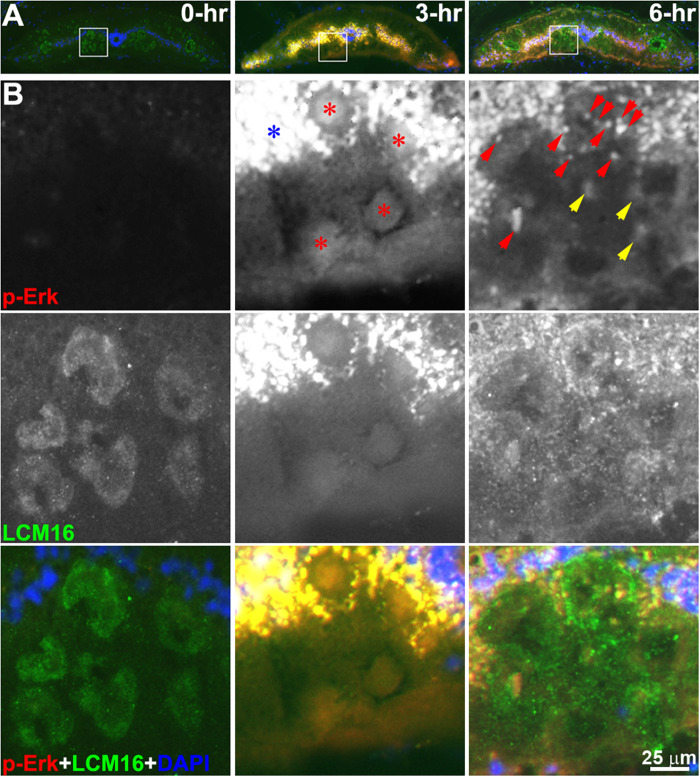
p-Erk staining becomes localized in granules by 6 h post-TX. **(A)** superimposed triple staining of cross sections at the level of the 6.5th gill in control (0-h) and transected (7th gill) spinal cords (3-h and 6-h). **(B)** enlarged squares in panel **(A)**. Two monochromic images in each column represent p-Erk and LCM16 labeling, respectively. The color images at the bottom is superimposed of p-Erk, LCM16 and DAPI staining. Diffuse p-Erk staining was found inside axons at 3 h post-TX (red stars), and in cells of the gray matter (blue star). Both granules (red arrowheads) and diffuse stain (yellow arrowheads) were found in axons of transected spinal cord at 6-h post-TX. Red, p-Erk; Green, LCM16; Blue, DAPI.

We examined changes in intra-axonal p-Erk-containing granules at 3 spinal cord locations (S1, S2, and S3 in Figure 3B) in previously transected spinal cords (Figure 5). LCM16 was used to identify axon profiles, and DAPI nuclear staining was used to identify cell bodies. Twelve animals were divided into 4 groups (untransected Ctrl, and 3, 6, and 24 h post-TX). Representative immunofluorescence images (Figure 5A) showed p-Erk labeling at 3 h in sections at S2. In addition to intra-axonal granules (red arrowheads), there were patches of more diffuse intra-axonal staining (yellow arrowheads). Immunolabeling for intra-axonal p-Erk was mostly diffuse at 3 h and replaced by granules at 6 h post-TX (Figure 6). For each frozen section, p-Erk-positive granules were counted at high magnification in six cross-sectional regions of axons (Figures 5Aa-f). The small-medium sized axons of the dorsolateral areas had far fewer p-Erk-positive granules than did the large Müller axons of the ventral columns and the Mauthner axons of the lateral columns. The small axons of the dorsal columns were not counted because they were mainly rostral-projecting and thus any movement of granules detected in them could not be assumed to be centripetally oriented. In addition, those axons do not stain prominently with LCM16. Nevertheless, the dorsal columns contained almost no p-Erk-positive granules.

Granules were counted in 4 serial sections taken from each of the three sampled spinal cord levels (S1, S2, and S3). Some intra-axonal granules were found inside areas of diffuse pErk staining and also were included in the count. Intra-axonal granules were rare in untransected control animals (2.2 ± 0.6, 1.9 ± 0.7, and 2.7 ± 0.9 per entire section at levels S1, S2, and S3, respectively). After TX, granules in large Müller and Mauthner axons increased earliest at S1, reached an approximately 32.7-fold peak increase by 6 h at S1 and 31.5-fold increase at S2, falling from those levels by 24 h. However, at S3, granule numbers were still rising at 24 h. This was true for both large and small-medium sized axons (Figure 5B). These findings suggest that Erk is activated (phosphorylated) near the lesion, and is translocated rostralward in granules.

### Fluorescence IHC Detection of p-Erk in Horizontal Sections of Transected Lamprey Spinal Cord

**FIGURE 7:**
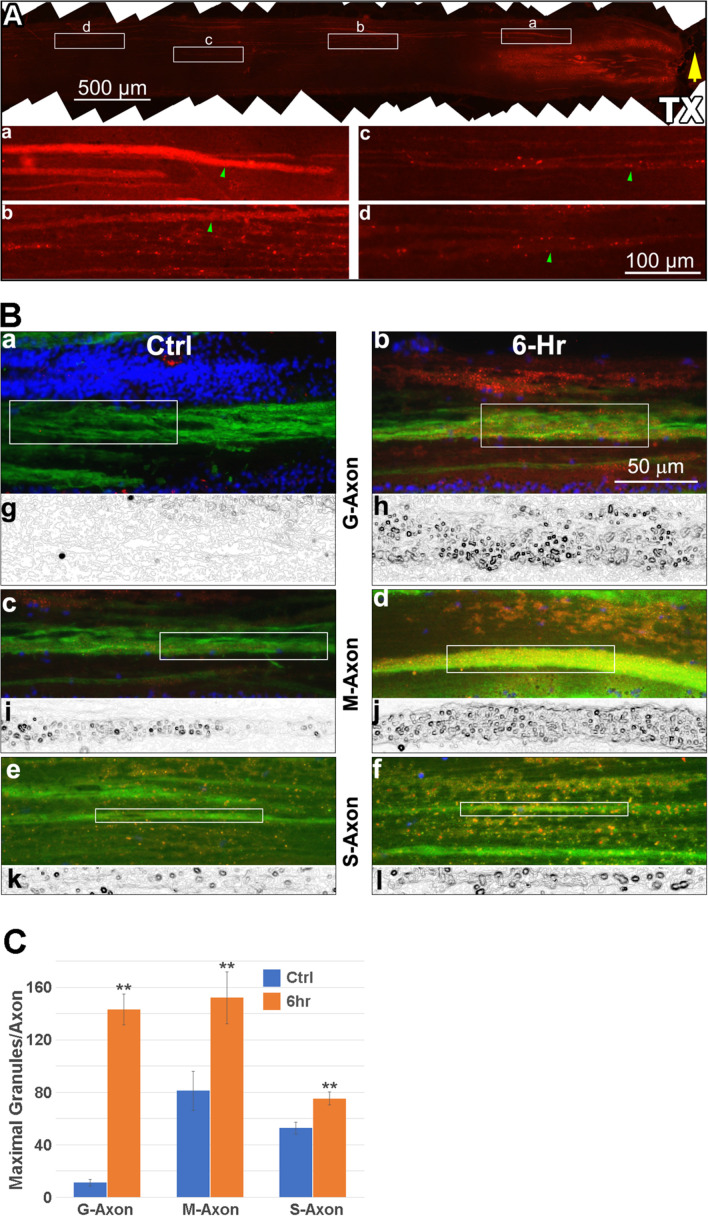
p-Erk staining is increased in axons after TX. Six spinal cords were transected at the level of the 7th gill, and 6 h later, immunolabeled with antibodies for p-Erk and neurofilaments (LCM16), followed by AF594- and AF488-labled secondary antibodies, respectively. Four untransected animals were used as controls. **(A)** a representative immunofluorescence image of p-Erk labeling in a horizontal section covering the spinal cord between the 4.5th and 7th gill (rostral is left). Rectangles **(a–d)**, shown enlarged below each frame, enclose 500 μm lengths of axon that are continuously present at maximum width within the section, and thus contain the maximum number of granules. The images illustrate the progressive reduction in p-Erk staining with distance rostral to the TX. Green arrowheads: 4 axons of similar caliber (10-11 μm width). **(B)** IHC for p-Erk in axons located at the -6th gill area, 1-2 mm from the site of TX at the 7th gill. **(a,b)**, giant axons (G-Axon, caliber > 30 μm); **(c,d)**, medium-caliber axons (**M-Axon**, 15-30 μm); **(e,f)**, small-caliber axons (**S-Axon**, < 15 μm) in spinal cords with **(6-h)** and without **(Ctrl)** TX, respectively. Red: p-Erk; Green: LCM16; Blue: DAPI. The boxes enclose 100 μm lengths of axon that are continuously present at maximum width within the section, and contain the maximum number of granules. **(g–l)**, binary images of the red channel in panels **(a–f)**, processed with the tool “Find Edges” (Adobe Photoshop CS4), to highlight countable round granules. **(C)** comparison between the numbers of p-Erk granules per 100 μm length of axon in spinal cords with TX *vs.* without TX. For each size category, the numbers of granules found in axons at 6 h post-TX are significantly greater than in the untransected control group (Mean ± SEM, *n* = 8-15 sections, **p < 0.01, Student’s *t*-test).

To further support the hypothesis that Erk is activated initially at the TX site and then retrogradely transported, we performed IHC on horizontal sections and determined the location of maximal p-Erk staining at 0, 3, 6 and 24 h post TX. The lamprey spinal cord is approximately 225 μm thick, yielding about 15 sections per length of spinal cord when sectioned at 15 μm (Dash line in Figure 2A). Small or medium caliber axons are observed in the dorsal-most 4-7 sections. The giant RS axons are encountered in the 8th-11th sections (Figure 2A). The distribution of p-Erk-containing granules along axons was studied by immunofluorescence. Horizontal sections of spinal cord encompassing the 4.5th to 7th gill (approximately 4.5 mm), were immunolabeled for p-Erk at 6 h post-TX. A representative section is seen at low magnification in Figure 7A. Immunofluorescence was diffuse in longitudinal profiles close to the TX site, consistent with the results in transverse sections. At higher magnification individual granules could be discerned (Figures 7A,a-d). Even within this length of spinal cord corresponding to S1, granule density was highest near the TX site. To confirm that these p-Erk-positive granules were inside axons we double-labeled the sections for neurofilament with LCM16 (Figures 7B,a-f). Statistical analysis showed that at 6 h post-TX, the number of p-Erk granules were significantly higher in giant, medium, and small-caliber axons than in corresponding sized axons of untransected spinal cords, and the difference was more extreme the larger the axons (Figure 7C). To eliminate a contribution to the findings from autofluorescence (Chen et al., 2015), we also performed chromogenic IHC in normal and transected spinal cord (data not shown). Serial horizontal sections of spinal cords 3 or 6 h post-TX, or from untransected controls, were incubated with p-Erk antibody, and visualized by biotinylated horseradish peroxidase (HRP). There was little or no p-Erk staining in sections of control spinal cord. Spinal cord TX elicited strong p-Erk staining at the TX site by 3 and 6 h.

### Spinal Cord TX Increases Vimentin mRNA and Protein Levels Rostral to the Lesion

**FIGURE 8:**
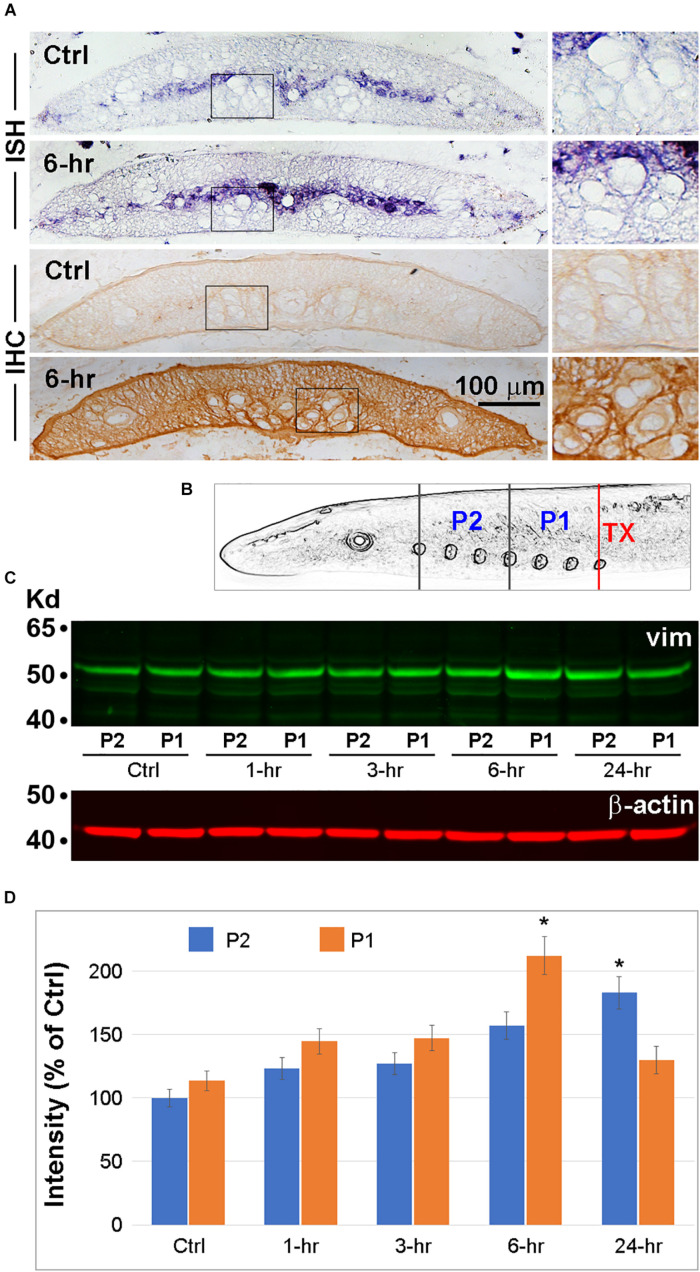
Vimentin expression is increased by spinal cord TX. **(A)** Transverse sections of spinal cord at the level of the 6th gill collected from either control animals or animals 6 h post-TX at the level of the 7th gill. **ISH** and **IHC** for vimentin were performed on sections close to the TX site. Each process was repeated twice in each of 4 animals/group. Right panels are magnified views of the corresponding boxed areas. **(B)** Schematic illustration of the locations of spinal cord lengths sampled for vimentin Western blotting. Six spinal cords/group at 5 time points **(Ctrl**, and **1**, **3**, **6**, and **24 h** post-TX at the 7th gill) were homogenized by sonication. Each spinal cord was divided into 2 pieces: P1 = 4th gill to 7th gill, P2 = 1st gill to 4th gill. **(C)** Western blotting was performed with mAbs against vimentin (1:1000, V6630, Sigma) and secondary antibody IRDye 800CW (926-32210; LI-COR). Loading controls (β-actin) are shown underneath. **(D)** Quantification of signals from **(C)** with ImageJ, after correction for background (Mean ± SEM, *n* = 3 animals/group. *p < 0.05 vs. control by ANOVA followed by Dunnett’s test).

In order to determine whether spinal cord TX increases the levels of vimentin mRNA in RS axons close to the injury, we performed ISH. Four control lampreys and four lampreys 6 h post-TX at the 7th gill, were killed and the body lengths between the 4th and 6th gill were cryo-sectioned. ISH was performed with a vimentin antisense probe. Other slides were used for chromogenic IHC with an anti-vimentin antibody. Both vimentin mRNA and protein were elevated in the transected spinal cords compared to controls. In the untransected spinal cord, vimentin mRNA and protein were present mostly in cell bodies of the gray matter and in the glial scaffold. No structures were labeled by the control sense probe. Intra-axonal levels were low (Figure 8A). Spinal cord TX increased extra-axonal vimentin mRNA and both intra-axonal and extra-axonal vimentin protein (Figure 8A). These findings were confirmed with Western blots. Thirty animals were separated into five groups (control, and transected at the 7th gill, with recovery for 1, 3, 6 or 24 h). Spinal cords between the TX site and the 1st gill were collected and cut into 2 pieces, P1 (caudal) and P2 (rostral) as shown in Figure 8B. Tissues were homogenized and separated in 10% SDS gel. A representative Western blot is shown in Figure 8C. Vimentin expression was significantly increased at 6 h (P1) and 24 h (P2) post-TX, respectively (Figure 8D).

### Vimentin Does Not Colocalize With p-Erk

**FIGURE 9:**
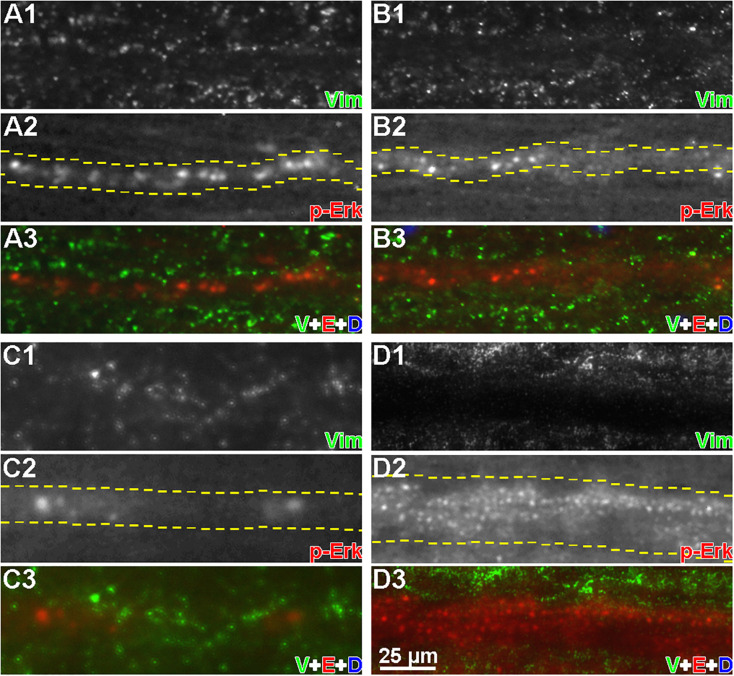
Vimentin and p-Erk are not colocalized in axons. Horizontal cryostat sections of spinal cords, each focused on a single axon, at 6 h post-TX were immunofluorescence labeled with anti-p-Erk and anti-vimentin antibodies. **(A1–D1)** show vimentin staining; **(A2–D2)** show p-Erk staining (white). Dashed lines indicate the axon boundaries; **(A3–D3)** show vimentin (green), p-Erk (red), and DAPI (blue) staining superimposed. **(A,B)** small caliber axons; **(C)** a medium caliber axon; **(D)** a large caliber axon. It was not possible to keep the entire length of axon in the plane of focus. Nevertheless, it can be seen that the vimentin stain was largely outside the axons, and not colocalized with p-Erk (*n* = 3 animals/group).

Axons of different calibers were examined by fluorescence microscopy in horizontal sections after double immunolabeling for p-Erk and vimentin (Figure 9). Linear arrays of p-Erk-positive granules (red) were gathered in regions of axon tracts. The absence of DAPI DNA staining in all triple-superimposed images (Figures 9A3-D3) indicates that the linear arrays are not in the gray matter. By contrast, vimentin-positive particles (green) were located outside the axons (Figure 9D), and did not colocalize with p-Erk.

### Dynein Colocalizes With p-Erk

**FIGURE 10:**
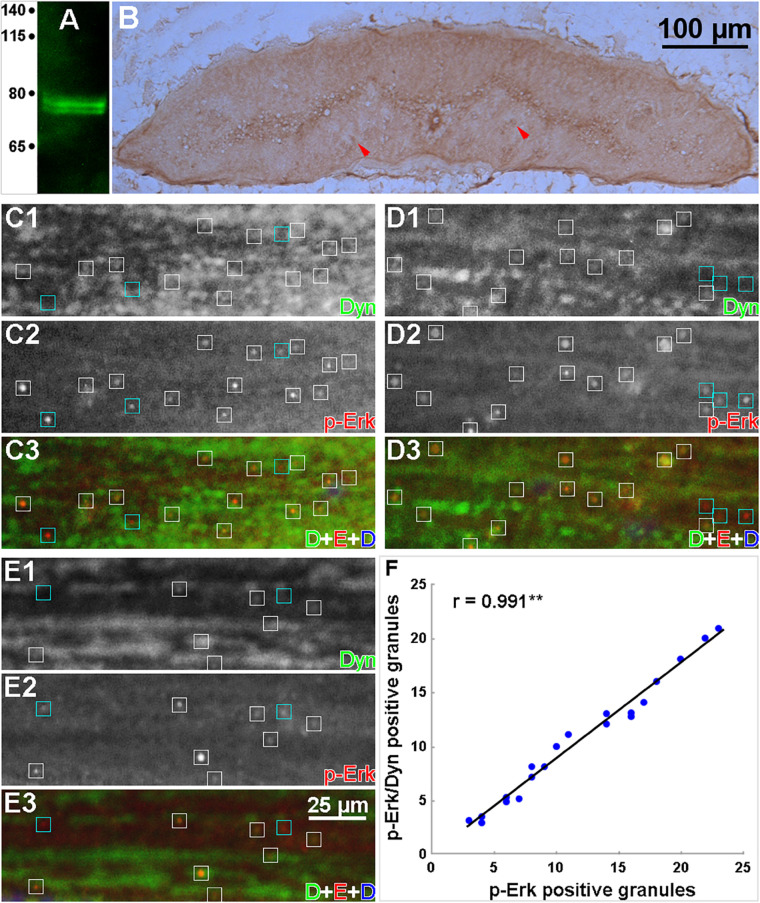
Colocalization of dynein and p-Erk in intra-axonal granules. **(A)** Western blot of proteins prepared from lamprey spinal cord, probed with an anti-dynein mAb. Two closely migrating bands were labeled at approximately 74 kDa. **(B)** IHC in a transverse section of normal lamprey spinal cord labeled with the anti-dynein mAb, showing diffuse dynein expression in both cell bodies and axons (red arrowheads). **(C–E)** data were collected from 2 experiments on 4 animals. Horizontal cryostat sections of spinal cords at 6 h post-TX were double-labeled immunofluorescently for p-Erk and dynein, and stained for DNA with DAPI. **(C1–E1)**, dynein staining; (**C2**-**E2),** p-Erk staining; **(C3–E3)**, dynein (green), p-Erk (red), and DAPI (blue) superimposed. **White boxes** enclose double-labeled granules; **Cyan boxes** enclose single-labeled (p-Erk) granules. Most p-Erk-labeled granules (88%) also are dynein-labeled. The absence of DAPI staining indicates that the granules shown are in axon tracts, not in areas with cell bodies. **(F)** fluorescent images captured in panels **(C–E)** were used for Pearson’s correlation analysis. Twenty areas (160 × 50 μm) were randomly selected for counting granules of p-Erk and dynein staining. Data analyzed by linear regression (SigmaStat) revealed a strong positive correlation between p-Erk and dynein labeling (**p < 0.001, *n* = 20). Because dynein is a universal retrograde molecular motor, it is widely distributed within the axons, and it may be difficult to intuit this colocalization by inspection without the statistical analysis.

Double-label IHC was used to determine whether the retrograde movement of p-Erk involves the molecular motor dynein. The specificity of the anti-dynein primary antibody (MAB1618, Millipore) for lamprey dynein was checked by Western blotting of protein isolated from normal lamprey spinal cord (Figure 10A). A double band was detected at approximately 74 kDa, similar to dynein in mammals (Pfister et al., 1996). IHC in transverse sections showed that dynein was present diffusely throughout the spinal cord (Figure 10B), including cell bodies in gray matter and axons. Horizontal sections from four spinal cords 6 h post-TX at the 7th gill were reacted with antibodies to dynein and p-Erk. Figure 10 illustrates the result in several small-caliber axons (Figures 10D,E) and one giant axon (Figure 10C). Approximately 88% of granules were double-labeled, indicating colocalization of p-Erk and dynein. Dynein is a universal retrograde molecular motor, so it is widely distributed in axons, and is associated with a large variety of cargos of different sizes, whereas only a small fraction of their cargo are the p-Erk-containing granules. This may make it difficult to intuit the colocalization of dynein and p-Erk-containing granules. To ameliorate this problem, in Figure 10, we placed boxes around the p-Erk-positive granules, white boxes for those also associated with dynein immunolabeling, and cyan boxes for those not associated with dynein immunolabeling. We then verified the colocalization of dynein with p-Erk by performing a Pearson’s correlation analysis. Twenty rectangular areas (160 × 50 μm) were randomly selected from 10 captured images to count the p-Erk-positive granules with and without dynein staining. The data were analyzed by linear regression. The strong positive correlation between p-Erk and dynein staining in granules is shown in Figure 10F (*y* = 0.902 *x* – 0.431, *r* = 0.991, *n* = 20).

### Axotomy Increases Phosphorylation of Erk in the Spinal Cord and of c-Jun in the Brainstem

**FIGURE 11:**
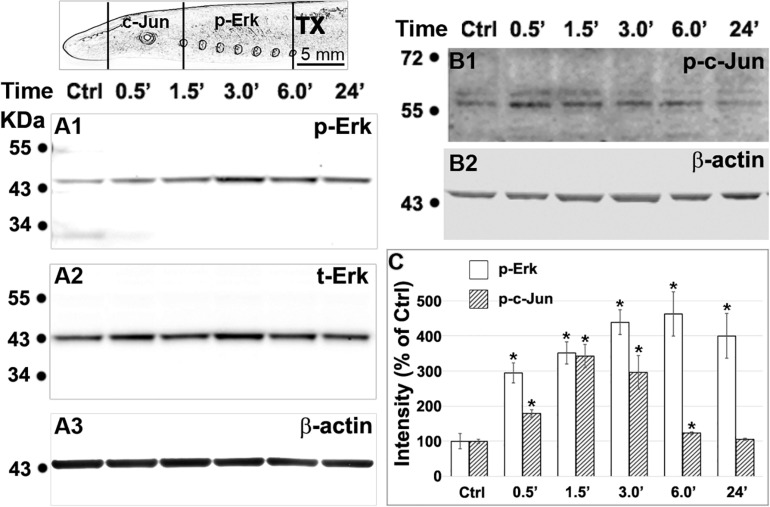
Axotomy increases phosphorylation of Erk in the spinal cord and c-Jun in the brainstem. Spinal cords and brains were pooled and homogenized (4 animals for each time point). **(A1,B1)**, western blotting performed with primary antibodies against p-Erk **(A1)** in the spinal cord and p-c-Jun **(B1)** in the brainstem (see top left scheme). **(A2)** the same membrane as in panel **(A1)** was probed for t-Erk. The sizes of p-Erk and t-Erk were not distinguishable (44 kDa). **(A3,B2)**, loading controls for panels **(A1,B1)**, respectively. **(C)** Phosphorylation of Erk in the spinal cord and of c-Jun in the brainstem, quantified with ImageJ after correction for background (Mean ± SEM, *n* = 5 for p-Erk, *n* = 4 for p-c-Jun; *p < 0.05 compared with control, ANOVA followed by Tukey Test).

To further elucidate the role of Erk in retrograde signaling of injury, after spinal cord TX, we evaluated the changes in p-Erk levels in the spinal cord, and c-Jun, a potential target of p-Erk, in the brainstem, where the spinal-projecting neurons are located. Twenty-four lampreys in six groups (control, and 0.5, 1.5, 3, 6, and 24 h post-TX) were used in these assays. Western blots showed that phosphorylation of both proteins appears at 0.5 h post-TX (Figure 11). Erk activation was dramatically increased even at 0.5 h, reaching a peak at 6 h but remaining significantly elevated at 24 h. Quantitation of t-Erk showed no significant increase post-TX. An increase of 120 ± 19% of control, observed at 6 h post-TX, was not statistically significant (one-way ANOVA). Phosphorylation of c-Jun also rose rapidly, reaching a peak at 1.5 h, but by 24 h, p-c-Jun had returned to normal levels. Although at 0.5 h, the increase in p-Erk was more dramatic than that of c-Jun, we cannot exclude that they rose in parallel, rather than that the rise of p-Erk in the spinal cord preceded that of c-Jun in the brain.

## Discussion

Several mechanisms have been proposed to explain how axons signal injury to their perikarya. Ions and biological macromolecules have been identified as retrograde injury signals, acting over seconds to minutes to days or even weeks (Ambron and Walters, 1996). In dorsal root ganglion (DRG) cells, complexes of Erk1/2 with other proteins have been postulated as retrograde injury signals (Hanz and Fainzilber, 2006). Much less is known about retrograde injury signals in the CNS, where assessment in mammals is complicated by several factors described in section “Introduction”. Here we report that at the site of spinal cord TX in the lamprey, activated Erk accumulates in RS axons near the site of injury, is translocated retrogradely to the perikarya in the brainstem, where c-Jun is activated. The activation of Erk is detected by 0.5 h post-TX, and lasts more than 24 h in the injured spinal cord. Because increased c-Jun activity also was detected by 0.5 h, and peaked by 1 h, whereas p-Erk levels peaked at 6 h, it was not possible to conclude that the Erk activation preceded that of c-Jun, even though at 0.5 h the increase in p-Erk was much greater than that of c-Jun. It is possible that the c-Jun activity in the brain was triggered at least in part by other factors, such as injury discharges that could lead to very rapid calcium-mediated c-Jun responses (Murphy et al., 1991; Cruzalegui et al., 1999).

### Lampreys Lack Erk1

In this study, antibodies to p-Erk (phosphorylated) and t-Erk (pan-specific) were used. In mammals, these antibodies do not distinguish Erk1 from Erk2 because they share 83% amino acid identity (Cargnello and Roux, 2011). In the present study, Western blots of CNS homogenates, using both antibodies, identified only a single Erk band. This was supported by DNA sequence analysis. A phylogenetic tree was constructed with 8 mammalian Erk genes, four of them Erk1, and another four of them Erk2. Four lamprey EST sequences were incorporated into the tree. Four ESTs (CO544767, CO548040, CO545630, and CO547047) (Pancer et al., 2004) belonged to the *erk2* clade, and one turned out to be a homolog of MAPKK. No Erk1 isoform was found. Thus, the sea lamprey appears to possesses only one isoform of Erk, Erk2. Because lampreys are considered the closest living relatives to the hypothetical primitive vertebrate ancestor, this is consistent with the suggestion of Buscà et al. (2015, 2016) that Erk2 represents the ancestral Erk.

### Erk as an Injury Signal

In the present study, Erk was activated at the TX site within 0.5 h post-TX, initially distributed diffusely in the cytosol of axons. Western blotting and IHC demonstrated that within 24 h, p-Erk was being translocated retrogradely in granules. The origins of the p-Erk are not known. Although in spinal cord white matter, levels of t-Erk increased close to the TX, this was much less dramatic and much slower than the increase in p-Erk. We conclude that the increase in t-Erk cannot explain the rapid increase in p-Erk, which must have been due primarily to activation of pre-existing Erk within the injured axon tip. The source of the Erk also is not known. It could derive from translation within the neuron itself, or be released by local cells near the injury, or from the blood. In sections of the TX site, increased p-Erk was found diffusely in both axons and nearby cells. Because Erk is abundant in human erythrocytes (Zennadi et al., 2012), it is possible that Erk released from the blood or injured nearby cells enters the unsealed axons, in a manner similar to that reported recently by this lab for fluorescent tracers (Zhang et al., 2018). After the first 6 h post-TX, p-Erk levels began to fall at the TX site, whereas they continued to increase at 24 h in the segments of spinal cord closest to the brain. This is consistent with retrograde transport of p-Erk away from the site of injury, but dephosphorylation at the injury site cannot be excluded.

### Erk’s Role in Axon Regeneration and Degeneration

Erk activation is a point of convergence for signaling pathways generated by a variety of axon growth inducers and noxious stimuli. Through phosphorylation of more than 100 different substrates, p-Erk regulates a broad array of cellular functions including proliferation, survival, apoptosis, motility, transcription, metabolism and differentiation (Ramos, 2008), in addition to axon regeneration. Erk’s role in neurite extension in response to growth factors is well-recognized. In peripheral nerve, Erk activation has been observed in transected sciatic nerve and ipsilateral DRG. This activation has been ascribed to the actions of a number of neurotrophic factors and cytokines (Sheu et al., 2000; Obata et al., 2003). Growth cone formation following axotomy in DRG or retinal ganglion cell (RGC) cultures (Chierzi et al., 2005) involves activation of Erk. N-cadherin, laminin and basic fibroblast growth factor (b-FGF) can activate Erk in embryonic chick retinal neurons and induce neurite outgrowth, which was inhibited by the Erk inhibitors, PD98059 and U0126 (Perron and Bixby, 1999).

Accumulated evidence suggests that Erk is involved in neuronal death. In cultured primary neurons or neuronal cell lines, cell death induced by multiple noxious stimuli can be prevented by pharmacological blockaded of the Erk pathway (Ho et al., 2008; Shirakawa et al., 2008; Chen et al., 2009). Consistent with a role for Erk in promoting cell death, the Erk pathway was implicated in hippocampal damage after traumatic brain injury, and in hyperglycemia-mediated cerebral damage (Zhang et al., 2007; Lu et al., 2008).

The mechanisms by which the Erk pathway might be involved in either axon regeneration or neuronal death are not completely understood. The intensity of Erk pathway activation could underlie the dual effects. Oxidant-induced neuronal death seems to require sustained Erk activation, while transient activation promotes cell survival or regeneration (Subramaniam and Unsicker, 2010). This agrees with our current finding that the heaviest p-Erk staining was found in the large-caliber axons of the ventral column (Figures 5Aa-d, 6). Their cell bodies in brainstem belong to the most poorly surviving, poorly regenerating identifiable RS neurons (I1, B1, B3, B4, and Mauthner) (Buchanan, 2001). Similarly, in mammals, large DRG neurons preferentially show Erk1/2 activation after sciatic nerve severing (Obata et al., 2003). The intense and persistent activation of p-Erk may be a cause of their selective vulnerability to apoptosis after axotomy. Erk activity can promote either intrinsic or extrinsic apoptotic pathways by induction of mitochondrial cytochrome *c* release or caspase-8 activation (Cagnol and Chambard, 2010). This is consistent with a previous report that after spinal cord TX in the lamprey, the extrinsic apoptotic pathway (caspase-8) is activated locally in the giant RS axons and retrogradely translocated to their cell bodies. Intraneuronal caspase-8 accumulation and cell death in these brainstem neurons was prevented by local treatment with the microtubule stabilizer Taxol (Barreiro-Iglesias et al., 2017). Whether the caspase and Erk pathways converge or are parallel mediators of retrograde neuronal death is not clear, since there exist Erk independent mechanisms by which caspase-mediated apoptotic pathway is activated (Sobrido-Camean and Barreiro-Iglesias, 2018).

### Dynein Colocalizes With p-Erk-Containing Granules, but Vimentin Does Not

Dynein-related retrograde transport is involved in signaling of axon injury to cell bodies (Rishal and Fainzilber, 2014). Axon injury activates Erk, and other MAPK (JNK, DLK) pathways. This is said to involve local protein translation of multiple constitutive components, including RanBP1, importin β1, vimentin, STAT3 and Luman, and is triggered locally by calcium waves (Tasdemir-Yilmaz and Segal, 2016). The locally translated proteins form a complex and are retrogradely transported to the cell bodies in granules *via* a dynein molecular motor, to activate nuclear transcription factors such as Elk1 (Perlson et al., 2005). In the present study, dynein indeed localized with p-Erk-containing granules.

Vimentin is reported to participate in the complex of retrograde Erk signaling (Perlson et al., 2005). The role of vimentin was clarified by experiments showing that when cleavage products of vimentin complexed with p-Erk they inhibited its dephosphorylation (Perlson et al., 2006). Vimentin is expressed in injured cultured DRG neurons (Willis et al., 2005), and in neurons during Alzheimer disease pathogenesis (Levin et al., 2009). Thus, in mammalian peripheral nerve, locally translated vimentin appears to play a role in the retrograde signaling of axon injury. However, until now, vimentin has not been reported in CNS neurons whose axons have been severed by spinal cord injury. In the present study, IHC showed that normal lamprey CNS axons contain a low background level of vimentin protein, but no intra-axonal vimentin mRNA was found by ISH. In whole-mounted lamprey brain, ISH with a vimentin probe showed widespread staining of small cells, mostly glia (Jin et al., 2016). Spinal cord TX resulted in an increase in vimentin mRNA and protein in axons near the injury site by 6 h post-TX. This increase in vimentin expression was transient, dropping to normal levels by 24 h post-TX. In a previous study, vimentin mRNA was not detected in axoplasm micro-aspirated from growing axon tips (Jin et al., 2016), suggesting that vimentin is not locally translated, but arrives at the injured RS axon tip either by transport from the perikaryon, or by diffusion from the extracellular space after release by injured glial cells. Moreover, vimentin did not colocalize with p-Erk in granules by IHC. The reason for the difference between these findings and those in mammalian peripheral nerve is not known. It may indicate a difference between axons in CNS and the periphery, or a species difference. It also is possible that the difference results from the different techniques employed to study the protein-protein interactions. Our colocalization study using double immunofluorescence IHC might be more precise than immune-coprecipitation performed in peripheral nerves, in which the exact source of vimentin is not established; it could be derived from axons, from Schwann cells, from serum or a combination (Rishal et al., 2010). We conclude tentatively that vimentin is not critical to the retrograde signaling of axon injury by p-Erk.

### Activation of c-Jun in the Brain After Spinal Cord Injury

c-Jun is one component of the activator protein-1 (AP-1) complex. Its typical upstream activators are the c-Jun amino-terminal kinases (JNKs)(Meng and Xia, 2011). The current study showed that spinal cord TX increases levels of p-c-Jun in the brain, and that this lagged behind the increase in p-Erk in the spinal cord during the first half hour post-TX. However, we did not establish a direct causal link between the retrograde movement of p-Erk and activation of c-Jun. Nor did we establish that the increase in c-Jun activation was specific to the axotomized RS neurons. A functional connection between Erk and c-Jun has been established by mutation studies in fission yeast, in which homologs of mammalian Erk and Jun act in concert to control yeast cell elongation immediately after division (Toda et al., 1991). Activation of c-Jun enhanced axonal outgrowth in rat sensory neurons (Lindwall et al., 2004), an effect (axonal outgrowth) consistent with that obtained with Erk1/2 activation. Inhibition of the ERK1/2 pathway suppressed Elk1 activation, and recruitment of c-Jun and Fra-2 to their promoters (Adiseshaiah et al., 2008). Moreover, constitutive activation of Erk increases c-Jun transcription and stability, which plays a role in the pathogenesis of human melanomas (Lopez-Bergami et al., 2007).

## Conclusion

The present study extends previous findings in mammalian peripheral nerve that the Erk pathway is involved in signaling nerve injury to the axons of a vertebrate CNS. Because *Petromyzon marinus* occupies a critical phylogenetic position between cephalochordates and gnathostomes, it is likely that the role of the Erk pathway in lamprey spinal axons will prove similar to its role mediating retrograde signals in mammalian CNS, including after spinal cord injury. In the lamprey spinal cord, p-Erk is associated with dynein but, unlike in mammalian peripheral nerve, not vimentin. Whether this is species-specific or a distinction between CNS and peripheral nerve is yet to be determined. Finally, the present findings may have molecular evolutionary significance. They support the notion that the lamprey lacks Erk1 isoforms. All four tested ESTs represent variants of the Erk2 isoform, and may have derived from a single ortholog by genomic duplication. Thus, the Erk1 of other vertebrate species probably evolved from an Erk 2 ancestral protein. Despite these new insights, there are limitations in interpreting static images of a dynamic process. Thus far, we have not obtained sufficient optical resolution to allow us to observe the movement of Erk-containing granules in the living spinal cord. However, this study helps to better understand the role of the Erk pathway in signaling axon injury in the lamprey spinal cord.

## Data Availability Statement

All datasets presented in this study are included in the article/supplementary material.

## Ethics Statement

The animal study was reviewed and approved by IACUC (protocol #: 4935) at the Temple University.

## Author Contributions

L-QJ did the ISH and carried out the molecular cloning and PCR. L-QJ and BJ did the immunohistochemistry. JH and L-QJ carried out the western blotting. L-QJ and MS carried out the data acquisition and analysis, drafted and prepared the final manuscript, and obtained the funding. All authors had full access to all the data in the study and take full responsibility for the integrity of the data and the accuracy of the data analysis.

## Conflict of Interest

The authors declare that the research was conducted in the absence of any commercial or financial relationships that could be construed as a potential conflict of interest.

## Footnotes

1 https://www.ncbi.nlm.nih.gov/
